# Analytical equation for outflow along the flow in a perforated fluid distribution pipe

**DOI:** 10.1371/journal.pone.0185842

**Published:** 2017-10-24

**Authors:** Huanfang Liu, Quanli Zong, Hongxing Lv, Jin Jin

**Affiliations:** 1 College of Water Conservancy and Architectural Engineering, Shihezi University, Shihezi, China; 2 College of Water Resource and Architectural Engineering, Northwest Agriculture and Forestry University, Yangling, China; Abdul Wali Khan University Mardan, PAKISTAN

## Abstract

Perforated fluid distribution pipes have been widely used in agriculture, water supply and drainage, ventilation, the chemical industry, and other sectors. The momentum equation for variable mass flow with a variable exchange coefficient and variable friction coefficient was developed by using the momentum conservation method under the condition of a certain slope. The change laws of the variable momentum exchange coefficient and the variable resistance coefficient along the flow were analyzed, and the function of the momentum exchange coefficient was given. According to the velocity distribution of the power function, the momentum equation of variable mass flow was solved for different Reynolds numbers. The analytical solution contains components of pressure, gravity, friction and momentum and reflects the influence of various factors on the pressure distribution along the perforated pipe. The calculated results of the analytical solution were compared with the experimental values of the study by Jin et al. 1984 and Wang et al. 2001 with the mean errors 8.2%, 3.8% and 2.7%, and showed that the analytical solution of the variable mass momentum equation was qualitatively and quantitatively consistent with the experimental results.

## Introduction

The perforated fluid distribution pipe is a typical type of dispensing equipment that can ensure that the main stream flows uniformly from the sidewall keyhole along the axial channel. Perforated pipes are widely applied in agriculture, the chemical industry, water supply and drainage, ventilation and other fields. In practical projects, the outlet of the lateral pipe might be a pipeline, spray nozzle or microspores. Because the total flow consists of separated multi-flows, the flow in the perforated pipe is also referred to as embranchment flow, in which the discharge, head loss and pressure distribution of the perforated pipes differ from those of non-perforated pipes. The flow characteristics of the perforated pipe are highly important for the pipeline design of sprinklers and drip irrigation projects and for applications in the chemical, dynamic, ventilation and environmental fields [1–8].

Flow distribution in a perforated fluid distribution pipe has been studied by a number of authors using the energy equation method [3,5,6,9–19]. Acrivos et al used one-dimensional flow equations to calculate the flow division in manifolds, and their results were applicable to a wide variety of combinations of channel dimensions, fluid velocities, physical properties, and pressure drops across the side ports[9]. Wu and Gitlin calculated the energy drop between outlets and the pressure distribution along a drip irrigation line with only 1% error by considering smooth pipes and using the Blasins equation for the friction coefficient[11].Warrick and Yitayew presented an alternative treatment that included a spatially variable discharge function as a component of the basic solution in lateral trickle systems[12,13].Following the derivation given by Shen developed an analytical solution to evaluate the effect of friction on flow distribution in both dividing and combining flow manifolds[14]. Scaloppi and Allen applied a differential approach to multiple outlet pipes with constant and continuously variable outflows and simulated the pressure distributions along uniform sprinkle systems, trickle irrigation laterals, manifolds, and gated pipes that considered the effect of ground slope and velocity head on the pipeline hydraulics[15]. Hathoot et al investigated the problem of a lateral pipe with equally spaced emitters and a uniform slope and estimated the head loss between emitters using the Darcy-Weisbach formula with variation in the Reynolds number, different zones on the Moody diagram, and a friction coefficient formula corresponding to each zone [16,17]. Jain et al developed a method for evaluating the lateral hydraulics using a lateral discharge equation approach and used a power equation to calculate the relationship between the inlet flow rate and inlet pressure head of the lateral [3]. Clemo developed a model of pressure losses in perforated pipes including the influence of inflow through the pipe walls and compared favorably with three experiments results [20]. A series of steady-state experiments were presented to study the stage discharge relationship for a porous pipe buried under loose laid aggregate [21–23]. Afrin et al studied the hydraulics of groundwater flow and porous pipe underdrains using a three-dimensional CFD model, and computed the discharge coefficient for the perforated pipe [24]. Maynes et al investigated the loss coefficient and onset of cavitation caused by water flow through perforated plates by an experiment [25].

Because the momentum conservation method can neglect the details of the flow and channel structure, the hole form and other factors contain a momentum exchange coefficient. This method was used to analyze the flow mechanism of a perforated fluid distribution pipe and formed the theoretical basis for the uniform fluid distribution [1,2,4,7,8,26–29]. Bassiouny and Martin analyzed mass and momentum balances for a flow element in both the intake and exhaust conduits based on one-dimensional flow equations [1,2]. By introducing a momentum equation, Jin et al studied a design method for determining the major parameters of branched pipe distributors in gas-solid fluidized beds for uniform gas distribution [4]. Kang and Nishiyama developed a lateral discharge equation to express the relationship between the discharge and pressure head at the inlet of a lateral using the finite element method [25,26]. Wang et al analyzed the friction and pressure recovery in porous pipe manifold coefficients and obtained an analytical solution of the momentum equation with varying mass and varying coefficients [7]. Wang et al introduced a general theoretical model to calculate the flow distribution and pressure drop in a channel with porous walls[8]. Yildirim and Agˇiraliogˇlu compared seven hydraulic methods to calculate the flow characteristics of a perforated pipe using micro-irrigation with special limited design conditions[29].

The above studies on the momentum conservation method are primarily based on the experimental results that were used to measure the friction coefficient and momentum exchange coefficient, and the solution of the theoretical model must be constrained to the condition of the constant coefficient. However, the flow in a perforated pipe occurs primarily under the condition of variable coefficients in practical projects. Therefore, an analytical solution for the variable coefficient is the key problem for flow distribution in a perforated fluid distribution pipe [8].

Another problem with the momentum method is that it neglects the influence of the slope on the flow in the process of establishing a variable mass momentum equation (considered only as a horizontal slope).According to mechanics, the momentum method considers only the influence of hydrodynamic pressure, friction and momentum changes on the flow but neglects the effect of gravity. For most perforated pipes used in micro-irrigation, the slope is not horizontal, and gravity might have a significant influence on the flow. Therefore, the gravity component should be introduced into the momentum equation of the variable mass to consider the influence of the slope.

The objectives of this study are to develop the momentum equation of variable mass flow using the momentum conservation method under the condition of a certain slope and to solve the momentum equation under different Reynolds numbers based on the velocity distribution of the power function. The analytical solution is suitable for flow in a perforated fluid distribution pipe, and for a perforated pipe with a small ratio between the length and diameter and flow is influenced by momentum and friction.

## Materials and methods

### Momentum equation of variable mass for outflow along the flow

The mass decreases along the flow in the porous tube flow or embranchment flow; thus, it is characterized as variable mass flow, and the pressure distribution is affected by momentum exchange, head loss and slope. The velocity of the flow decreases with changes in mass because of the holes in the sidewall; thus, the momentum changes along the flow, and a certain amount of kinetic energy is converted into the pressure head, which causes the pressure to increase along the flow. The friction produces pressure losses that cause the pressure to decrease, and at the same time, the flow might produce rough waves at the outlet of the hole, which can increase the energy losses. The head change induced by the slope of the pipe has a significant effect on the pressure distribution[7]. Therefore, it is necessary to simultaneously consider these influence factors if the pressure change is maintained in a certain range.

**Fig 1:**
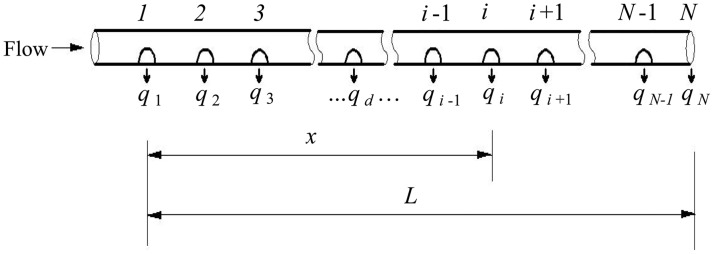
Schematic of pipeline with multiple outlets.

Fig 1 shows a schematic of the pipeline with multiple outlets. In Fig 1, the perforated tube is closed at the end of one side and the cross-sectional area is the same along the flow, which has one row of radial holes.

The following assumptions are applied: (i) the flow is one-dimensional; (ii) the fluid is incompressible; (iii) the velocity is 0 at the closed end; (iv) the environmental pressure is constant, and the flow through the holes in the sidewall is free flow; (v) the holes are vertical to the axis, and the distance and size of the holes are all uniform; (vi) the size of the cross-section is constant, and the slope is uniform along the flow; and (vii) based on these assumptions, the discharge of the holes depends on the pressure; i.e., the distribution of the discharge of the porous tube pipes depends on the pressure distribution.

**Fig 2:**
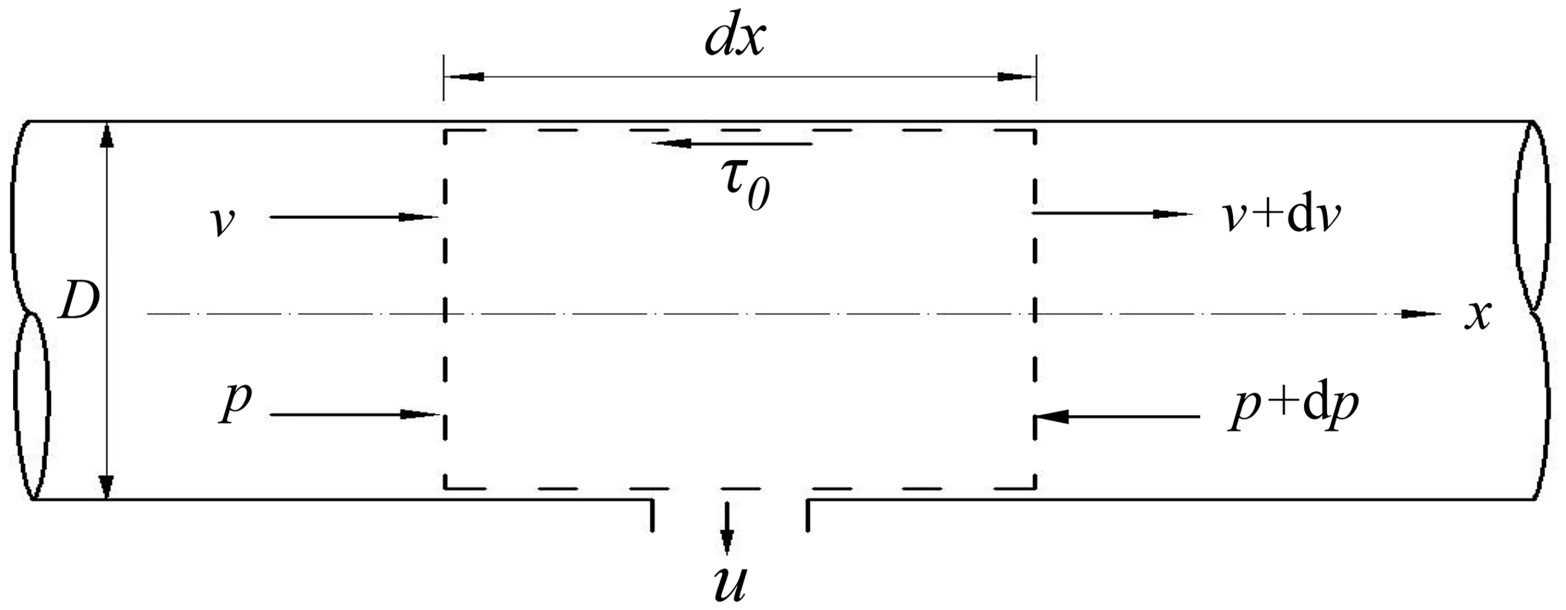
Control volume of varying mass flow.

For a flow of variable mass in the perforated pipe, we select a micro-control volume, as shown in Fig 2. We select the micro-interval d*x* along the *x*-axis (d*x* can contain several distances of holes), and all holes in an infinitesimal section have a uniform velocity, namely, the velocity of point *x*. Under this condition, we can establish the equation of continuity and momentum for the infinitesimal section, and the momentum equation of the variable mass is generally expressed as follows [30]:

$$\frac{1}{\rho }\frac{dp}{dx}+\frac{\lambda }{2D}V^{2}+2kV\frac{dV}{dx}=g\;I$$ (1)

where *p*—hydrodynamic pressure, N.m^-2^;*ρ*—density of the fluid, kg.m^-3^;*V*—velocity of the flow, m.s^-1^;*D*—tube diameter, m;*λ*—friction coefficient along the flow; *k*—momentum exchange coefficient; *g*—acceleration of gravity, m.s^-2^; and *I*—the slope of the tube.

The rationality and convenience of the momentum equation means that it can neglect the details of the flow and modify the effect of the diversion and vortex via the momentum exchange coefficient. Therefore, the momentum equation of the variable mass, which is the basis of the perforated pipe problem, is the theoretical model for practical engineering problems and can be used to calculate and analyze the flow mechanism and variation law of the perforated pipe. Because the momentum exchange coefficient *k* and the friction coefficient *λ* are functions of the velocity, Eq (1) contains a serious nonlinearity. The nonlinear equation displays three difficulties: (i) the variation law of the friction coefficient *λ* with the Reynolds number and system structure and the difference from the law of *λ* in the smooth pipe; (ii) the variation law of the momentum exchange coefficient *k* with the momentum and velocity of the fluid; and (iii) the solution of the nonlinear equation.

The critical problem in solution of the nonlinear differential equation lies in how to analyze and solve the equation while considering the peculiar properties of the embranchment flow. Based on previous results, this article investigates the variation law of the momentum exchange coefficient *k* and the friction coefficient *λ* by applying the theoretical and experimental methods simultaneously and subsequently solving the momentum equation of the variable mass.

### Velocity distribution in the perforated pipe

The flow in the perforated pipe is a variable discharge flow. Generally, the discharge varies among different holes; thus, the variation of the discharge is a complex function of the *x*-axis along the flow. The tube diameter *D* is a constant in the equal section tube, and the velocity is proportional to the discharge. Therefore, the velocity distribution can be assumed to be a power function [29]:

$$V=\frac{Q_{X}}{A}=\;\frac{Q_{0}}{A_{0}}{\left(1-\frac{x}{L}\right)}^{z}=V_{0}{\left(1-\frac{x}{L}\right)}^{z}$$ (2)

where *Q*_*x*_—discharge through the *x*-section, m^3^.s^-1^;*Q*_*0*_—total discharge (entrance discharge), m^3^.s^-1^;*L*—length of the perforated pipe, m;*V*_*0*_—velocity at the entrance, m.s^-1^;*A*_*0*_—size of the cross-section,*A*_0_ = 1/4π*D*^2^, m^2^; *X* = *x/L*; and *z*—an exponent.

**Fig 3:**
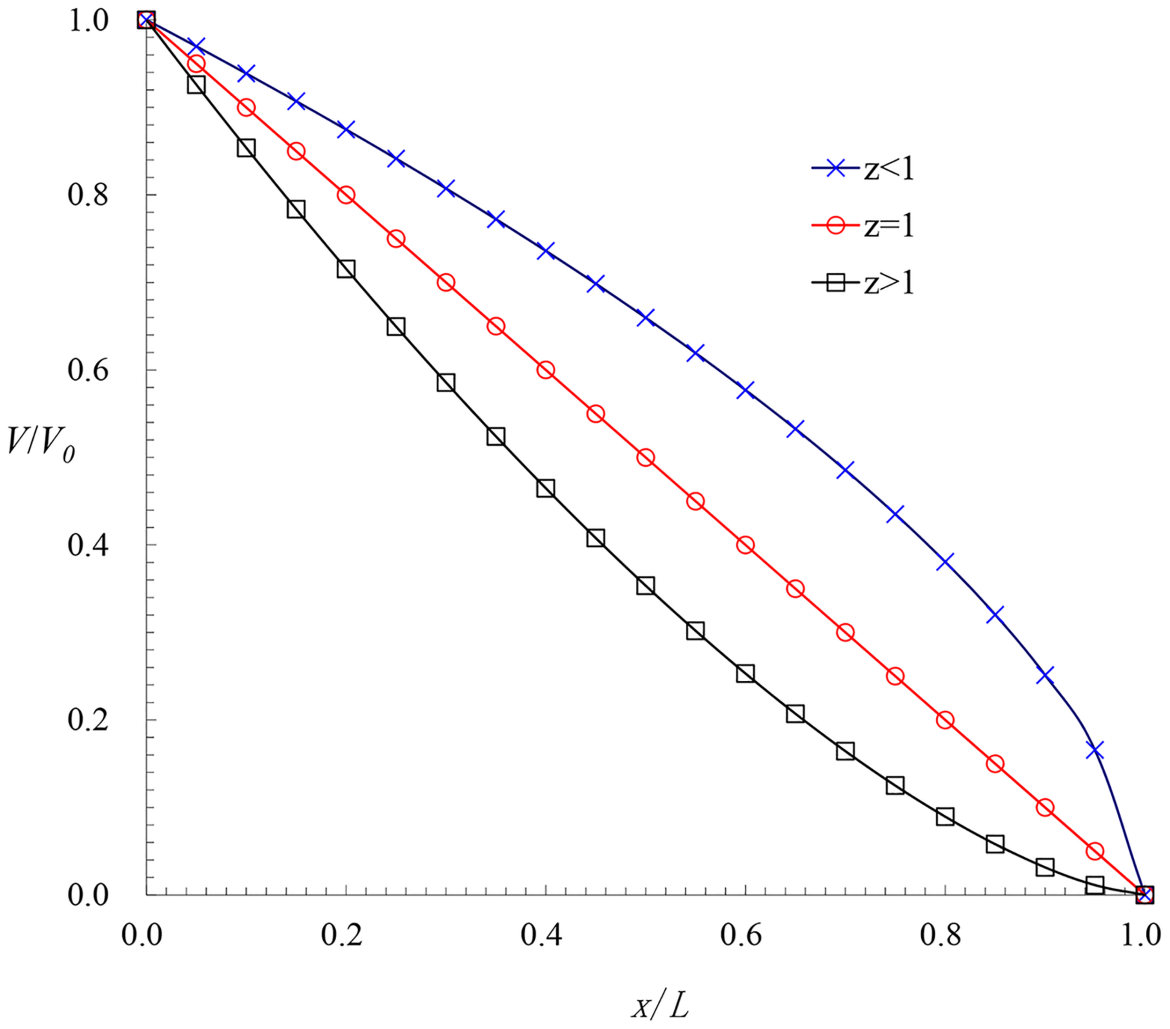
Dimensionless velocity profile.

Three types of velocity distributions occur with variation of the exponent *z*, as shown in Fig 3.

1. *z*<1; This situation corresponds to momentum-controlled flow, and in this condition, the momentum force is greater than the friction, and the velocity increases gradually along the flow.
2. *z* = 1; In this condition, the pressure term equals to the viscouse term. Thus, the hydrodynamic pressure and discharge of the holes are uniform along the flow, and the variation of velocity is linear, similar to the assumptions of the previous results. Strictly speaking, the discharge varies among different holes, but for uniform irrigation, the discharge of the holes should be uniform. Therefore, we quote the previous assumptions for the relative deviation confined to a smaller range (e.g., *q*_v_≤0.2 [26].
3. z>1; This situation corresponds to friction-controlled flow. In this condition, the friction is greater than the momentum force, and the velocity decreases gradually along the flow.

In equal-section porous pipes, the exponent in Eq (2) should be related to the opening ratio *η* (the area ratio of holes to the entire sidewall), the length-diameter ratio *E* (the length to the tube diameter), and the slope *I*. The following assumptions can be made: the relative deviation of the discharge from different holes becomes smaller, and the exponent *z* might reach approximately 1 and vice versa.

When *x* = 0.5*L*, *Q*_*0*.*5*_ = *Q*_*0*_·0.5^*z*^is derived according to Eq (2), and the calculation formula for the exponent *z* is expressed as follows:

$$z=\frac{1}{\text{ln}0.5}\cdot \;\text{ln}(\frac{Q_{0.5}}{Q_{0}})$$ (3)

where *Q*_*0*.*5*_is the discharge of the *L*/2-section and is equal to the algebraic sum of the discharge of the holes from *x* = 0.5*L*to x = *L*, namely:

$$Q_{0.5}=\sum_{j=N/2}^{N}q_{j}=\;Q_{0}-\sum_{j=1}^{N/2}q_{j}$$ (4)

where *j*—serial number of the hole (arrayed in order from the tube entrance to the end);*q*_*j*_—discharge of hole number *j*; and *N*—total number of holes in the perforated pipe.

According to the experimental discharge data and Eqs (3) and (4), the exponent *z* can be calculated, and the effect of all dimensionless numbers on the exponent *z* can be analyzed.

According to Eq (3), if *Q*_*0*.*5*_ = 0.5*Q*_*0*_, the algebraic sum of the discharge of the former half of the pipe is equal to the latter, and *z* = 1, which indicates that the discharge of the holes is uniform in the former and latter. Strictly speaking, the discharge is different, but the relative deviation is smaller under the condition of*Q*_*0*.*5*_ = 0.5*Q*_*0*_; thus, the equivalent outflow can be approximated. If*Q*_*0*.*5*_<0.5*Q*_*0*_, the discharge in the former half is more than that in the latter half, and *z*>1, which indicates that the outflow discharge is gradually reduced. If*Q*_*0*.*5*_>0.5*Q*_*0*_, the former is less than the latter, and *z*<1, which indicates that the outflow discharge increases gradually.

When the effects of the slope *I*, length-diameter ratio *E* and opening ratio *η* on the exponent *z* are mutually independent, the formula for the exponent *z* containing these dimensionless numbers can be expressed as follows [30]:

$$z=\varphi_{1}(I)\varphi_{2}(\;E)\;\varphi_{3}(\;\eta \;)$$ (5)

Where *E* = *L*/*D* is the ratio between length and diameter; and *η* = *A*_1_/*A*_2_ = (*Nd*^2^)/(4*DL*)is the opening ratio, which is the ratio of the total perforated area *A*_1_ = *N*·1/4π*d*^2^ to the entire internal face *A*_2_ = π*DL*.

First, if the opening ratio *η* and length-diameter ratio *E* are constant, the model fits the relationship of *I*~*z*. If the opening ratio is constant, the model fits the relationship of *E*~*z*, according to the *I*~*z* relationship. Finally, the model fits the relationship of *η*~*z*, considering the comprehensive influence of *I* and *E*, and the empirical formula of the exponent *z* can be expressed as follows:

$$z=(1-27\;I)\;(1+\;9\times 10^{-5}\;E)\;(1+0.036\;\text{ln}\frac{\eta }{\eta_{0}}\;)$$ (6)

where the range of *I* is -0.001–0.009, *E* is 286–2000,*η*_0_ = *d*_0_^2^/(2*D*_0_*S*_0_), and *d*_0_, *D*_0_ and *S*_0_ are, respectively, the diameter of the hole, the diameter of the tube and the hole distance. In this article, *d*_0_ = 0.0012m,*D*_0_ = 0.035m,*S*_0_ = 0.30m,*η*_0_ = 6.8571×10^−5^, and the range of *η*/*η*_0_ is 0.0625–2.

The exponent *z* can be calculated in different conditions according to Eq (6). Eq (6) indicates that the exponent *z* decreases with *I* but increases with *E* and *η*. In actual engineering projects, the exponent *z* should be determined according to the comprehensive influence of *I*, *E* and *η*, which indicates that to ensure that the exponent *z* reaches 1, the good best approach is to adjust *I*, *E* and *η*.

### Variation of the resistance coefficient *λ*

For the perforated pipe, the friction coefficient is related to the sidewall roughness and the tube structure in addition to the Reynolds number. In actual engineering projects, the Reynolds number decreases with the discharge along the flow (maximum at the entrance, 0 at the end). Therefore, the friction coefficient increases along the flow. If the flow pattern is smooth turbulent flow (*R*e<10^5^) at the entrance, the pattern at the end is laminar flow. In fact, laminar flow always exists at the end of the perforated pipe. In the solution process, if the formula of the friction coefficient*λ*_0_ is uniform along the entire pipe, then the calculated value of *λ* at the end might be less than the actual value, and the deviation will be highest at the closed end, which can affect the pressure distribution at the end, especially at a closed end. As such, *χ* is introduced to reflect this effect in the solution process of the variable mass momentum equation, i.e., *λ* = *χλ*_0_, where *λ*_0_is the friction coefficient in the smooth pipe, and *χ* is the modified coefficient.

If*R*e_0_<2000, the flow pattern of the entire pipe is laminar flow, the friction coefficient does not need to be modified, and *χ* = 1.0.

If*R*e_0_>2000, the section from the entrance to the approximate *R*e = 2000 point is turbulent flow and the other is laminar flow. The range of the laminar flow is related to*R*e_0_ (Reynolds number at the entrance) and the length-diameter *E*. In this condition, *χ*>1.0, and the range is 1.1–1.5. As a result, *χ* = 1.3 is always used to simplify the calculation process.

Different formulas of the friction coefficient exist for different values of *R*e. Thus, in the solution process, judgment of the flow pattern (*R*e) occurs first, and the proper formula is selected according to the value of *R*e.

### Momentum exchange coefficient *k*

The derivation of the variable mass momentum equation indicates that the momentum exchange coefficient is a modification of the momentum component, which is contained by the outflow. The velocity deviation between the entrance and the outlet of the hole is caused by the velocity component, which is contained by the outflow; i.e., the relative deviation ratio of the mainstream momentumΔ*V*^2^/*V*^2^ is equal to the ratio of the momentum component contained by the outflow to the total mainstream momentum. Because the momentum change is related to the vortex, friction, and momentum components contained by the outflow, the modified coefficient *β* should be used, namely, *β(*Δ*V*^2^)/*V*^2^. If the momentum variation ratio of the first hole is *α*, the friction coefficient *k* can be expressed as follows [31]:

$$k=\;\alpha +\;\beta \frac{\Delta V^{2}}{V^{2}}$$ (7)

When the velocity distribution of the mainstream is given, we can take the derivative of the relative momentum deviation and perform integration of this derivative to derive the functional expression of the relative momentum deviation, and finally, the general formula can be expressed as follows:

$$k=\alpha +\;2\beta \text{ln}\frac{V}{V_{0}}$$ (8)

The coefficients of *α* and *β* are constant. At the entrance, *x* = 0,*V* = *V*_0_, and *k* = *α*. At the closed end, *x* = *L*,Δ*V*^2^/*V*^2^ = -1, and *k* = 0.5.

According to Δ*V*^2^/*V*^2^ = -1and Eq (7), *k* = *α*−*β* can be derived, which is the momentum exchange coefficient of the last hole, and (*α*−*β*) applies at the closed end. According to previous results [14, 28], *β*≌0.15and*α*≌0.65, and these values are used in this article. This analysis indicates that the coefficients *α* and *β* have definite physical meanings based on the law of conservation of momentum, which is an advanced point in this article.

Combining Eqs (2) and (8), the formula of *k* can be expressed as follows:

$$k=\alpha +\;2z\beta \text{ln}(1-\;\frac{x}{L})$$ (9)

## Results and discussion

### Solution of the momentum equation

The process of establishment and derivation of the theoretical model includes the two variable parameters *λ* and *k* in Eq (1). The functions of *λ* and *k* have been given, and the momentum equation can be solved. By combining Eqs (8) and (1), Eq (10) can be derived:

$$\frac{1}{\rho }\frac{dp}{dx}+\frac{\lambda }{2D}V^{2}+\;(\alpha +\;2\beta \text{ln}\frac{V}{V_{0}})\frac{dV^{2}}{dx}=gI$$ (10)

Combining Eqs (10) and (2), Eq (11) can be derived:

$$\frac{1}{\rho }\frac{dp}{dx}+\frac{\lambda }{2D}V_{0}{}^{2}{\left(1-\;\frac{x}{L}\right)}^{2z}+V_{0}{}^{2}\;\left[\alpha +\;2z\beta \text{ln}(1-\;\frac{x}{L})\right]\frac{d(1-x/L{)}^{2z}}{dx}=gI$$ (11)

In actual engineering projects, the friction coefficient *λ* is variable, which affects the pressure distribution. However, *λ*_0_ is a function of the Reynolds number, and different formulas apply in different Reynolds number ranges. To derive the analytical solution under different conditions, we should investigate different ranges of Reynolds numbers.

#### *R*e<2000

According to *λ* = 64*χ/R*e = 64*νχ/*(*DV*) = 64*νχ/*(*DV*_0_(1-*x/L*)^*z*^)and Eq (11),

$$\frac{1}{\rho }\frac{dp}{dx}+\frac{32\nu \chi }{D^{2}}V_{0}{\left(1-\frac{x}{L}\right)}^{z}+V_{0}{}^{2}\;\left[\alpha +\;2z\beta \text{ln}(1-\frac{x}{L})\right]\frac{d(1-x/L{)}^{2z}}{dx}=gI$$ (12)

Integrating Eq (12) from 0 to *x* and using the dimensionless method, we obtain

$$Eu=\frac{gLI}{V_{0}{}^{2}}X-\frac{32\nu \chi L}{(z+1)D^{2}}V_{0}\left[1-{\left(1-X\right)}^{z+1}\right]+\alpha \left[1-{\left(1-X\right)}^{2z}\right]\;-2z\beta \left[{\left(1-X\right)}^{2z}\;\text{ln}(1-X)+\frac{1-{\left(1-X\right)}^{2z}}{2z}\right]$$ (13)

Or,

$$Eu=\frac{gIL}{V_{0}{}^{2}}X-\;\frac{32\chi E}{(z+1){\text{Re}}_{0}}\left[1-{\left(1-X\right)}^{z+1}\right]+\alpha \left[1-{\left(1-X\right)}^{2z}\right]\;-2z\beta \left[{\left(1-X\right)}^{2z}\;\text{ln}(1-X)+\frac{1-{\left(1-X\right)}^{2z}}{2z}\right]$$ (14a)

Where *E*_u_ = (*p*_x_-*p*_0_) /*ρV*_0_^2^,*X* = *x*/*L*,*E* = *L*/*D*, and*R*e_0_ = *V*_0_*D*/*ν*.

If *X* = 1, *Eu* contains the infinitive of the “0·∞” format, and in this condition, the limit value of *Eu* is equal to the value of *Eu*, namely,

$${Eu|}_{X=1}=\underset{X\to 1}{\text{lim}}Eu=\frac{gIL}{V_{0}{}^{2}}+\;\alpha \;-\;\beta -\;\frac{32\chi E}{(z+\;1)\;{\text{Re}}_{0}}$$ (14b)

#### 2000*≤R*e<10^5^

According to *λ* = 0.3164*χ*/*R*e^0.25^ = 0.3164*χ*/(*VD/ν*)^0.25^ and Eq (11),

$$\frac{1}{\rho }\frac{dp}{dx}+\frac{0.1582\chi \nu^{0.25}}{D^{1.25}}V_{0}{}^{1.75}{\left(1-\frac{x}{L}\right)}^{1.75z}+V_{0}{}^{2}\;\left[\alpha +\;2z\beta \text{ln}(1-\frac{x}{L})\right]\frac{d(1-x/L{)}^{2z}}{dx}=gI$$ (15)

Integrating the Eq (15) from 0 to *x* and using the dimensionless method, we obtain

$$Eu=\frac{gIL}{V_{0}{}^{2}}X-\frac{0.1582\chi E}{(1.75z+1){\text{Re}}_{0}{}^{0.25}}\left[1-{\left(1-X\right)}^{1.75z+1}\right]+\alpha \left[1-{\left(1-X\right)}^{2z}\right]\;-2z\beta \left[{\left(1-X\right)}^{2z}\;\text{ln}(1-X)+\frac{1-{\left(1-X\right)}^{2z}}{2z}\right]$$ (16a)

And,

$${Eu|}_{X=1}=\underset{X\to 1}{\text{lim}}Eu=\frac{gIL}{V_{0}{}^{2}}+\;\alpha \;-\;\beta -\;\frac{0.1582\chi E}{(1.75z+\;1)\;{\text{Re}}_{0}{}^{0.25}}$$ (16b)

#### 10^5^*≤R*e<10^7^

According to *λ* = 0.13*χ*/*R*e^0.172^ = 0.13*χ*/(*VD/ν*)^0.172^ and Eq (11),

$$\frac{1}{\rho }\frac{dp}{dx}+\frac{0.065\chi \nu^{0.172}}{D^{1.172}}V_{0}{}^{1.828}{\left(1-\frac{x}{L}\right)}^{1.828z}+V_{0}{}^{2}\;\left[\alpha +\;2z\beta \text{ln}(1-\frac{x}{L})\right]\frac{d(1-x/L{)}^{2z}}{dx}=gI$$ (17)

Integrating Eq (15) from 0 to *x* and using the dimensionless method, we obtain

$$Eu=\frac{gIL}{V_{0}{}^{2}}X-\frac{0.065\chi E}{(1.828z+1){\text{Re}}_{0}{}^{0.172}}\left[1-{\left(1-X\right)}^{1.828z+1}\right]+\alpha \left[1-{\left(1-X\right)}^{2z}\right]\;-2z\beta \left[{\left(1-X\right)}^{2z}\;\text{ln}(1-X)+\frac{1-{\left(1-X\right)}^{2z}}{2z}\right]$$ (18a)

And,

$${Eu|}_{X=1}=\underset{X\to 1}{\text{lim}}Eu=\frac{gIL}{V_{0}{}^{2}}+\;\alpha \;-\;\beta -\;\frac{0.065\chi E}{(1.828z+\;1)\;{\text{Re}}_{0}{}^{0.172}}$$ (18b)

The analytical solutions of the variable mass momentum equation are obtained in different ranges of Reynolds numbers according to the theory of power rate distribution, similar to Eqs (14a), (16a) and (18a). These equations contain the pipe slope *I*, the length-diameter ratio *E* and flow parameter*R*e_*0*_. At the same time, three dimensionless parameters (*E*_u_ = (*p*_x_-*p*_0_) /*ρV*_0_^2^,*F*r = *V*_0_/(*gL*)^0.5^, and*R*e_0_ = *V*_0_*D*/*ν*) are contained in these equations, which indicate that the major forces for porous flow are hydrodynamic pressure, gravity and friction. These three forces are interrelated, and all impact the porous flow. The three equations all contain the *Eu* number on the left-hand side of the equations, and *Eu* is the pressure item that reflects the variation of the hydrodynamic pressure. The right side of the three equations contains four items: gravity, friction and two momentum items. The first is the gravity item that reflects the influence of the slope *I* on *Eu*. The second item is the friction item that reflects the influence of the friction coefficient *λ* on *Eu*. The third and fourth items are both momentum terms that reflect the influence of momentum variation on *Eu*. In the three equations, the other items are the same, except for the second item, which instead reflects the influence of the Reynolds number on the drag coefficient *λ*. If the slope *I* and length-diameter ratio *E* are constant, the effect of the Reynolds number on the pressure distribution of the porous pipe can be derived. In the process of engineering design, the length-diameter ratio can be derived if the discharge and slope are given. For built projects, the three equations are used to assess the working condition of the perforated pipes and the rationality of the projects.

According to analysis of the analytical solutions for the momentum equation, the influence of the gravity, friction and momentum items on the *Eu* (hydrodynamic item) is unanimous from the point of view of quality in Eqs (14a), (16a) and (18a). Generally, we always consider the condition 2000≤*R*e<10^5^; thus, the effect of many factors on the pressure distribution of the porous pipe is analyzed based on Eq (16a).

The right side of Eq (16a) contains four items. The first is the gravity item that changes with the value of *X*. If *X* = 0, this term is equal to 0, and if *X* = 1, it is equal to *gIL*/*V*_0_^2^, which indicates that the effect of gravity on the pressure distribution is linear and that the gradient is related to the slope and length of the porous pipe. The second item is the friction item, which also changes with the value of *X*. According to the friction item, the effect of friction increases and the hydrodynamic pressure difference decreases as the length-diameter *E* increases or the Reynolds*R*e_0_ decreases, and vice versa. The third and fourth items are momentum items that change with the value of *X*. If *X* = 0, the result of third and fourth items is equal to *α*, and when *X* = 1, it is equal to (*α*−*β*). The variation indicates that the momentum exchange is variable, with a maximum at the entrance and a minimum at the closed end. Using the same type with the same reasoning, the same conclusions can be derived under the conditions of *R*e<2000 and 10^5^≤*R*e<10^7^. In the process of engineering design, if the slope is given, we can adjust the length-diameter and the Reynolds number to reflect the influence of the gravity, momentum and friction balance, and in this condition, the hydrodynamic pressure distribution tends toward a uniform distribution, and the discharges of the various holes are the same.

### Influence of slope on the pressure distribution

We verify the validity and accuracy of the analytical solution for the variable mass momentum equation according to the experimental results from the micro-pressure porous pipe. Generally, we always consider the condition of 2000≤*R*e<10^5^ and calculate the analytical solution based on Eq (16). To simplify the calculation process, *α* = 0.65 and *β* = 0.15 are selected. For the exponent *z*, we first select *z* = 1 and verify according to Eq (6) and the actual parameters.

**Fig 4:**
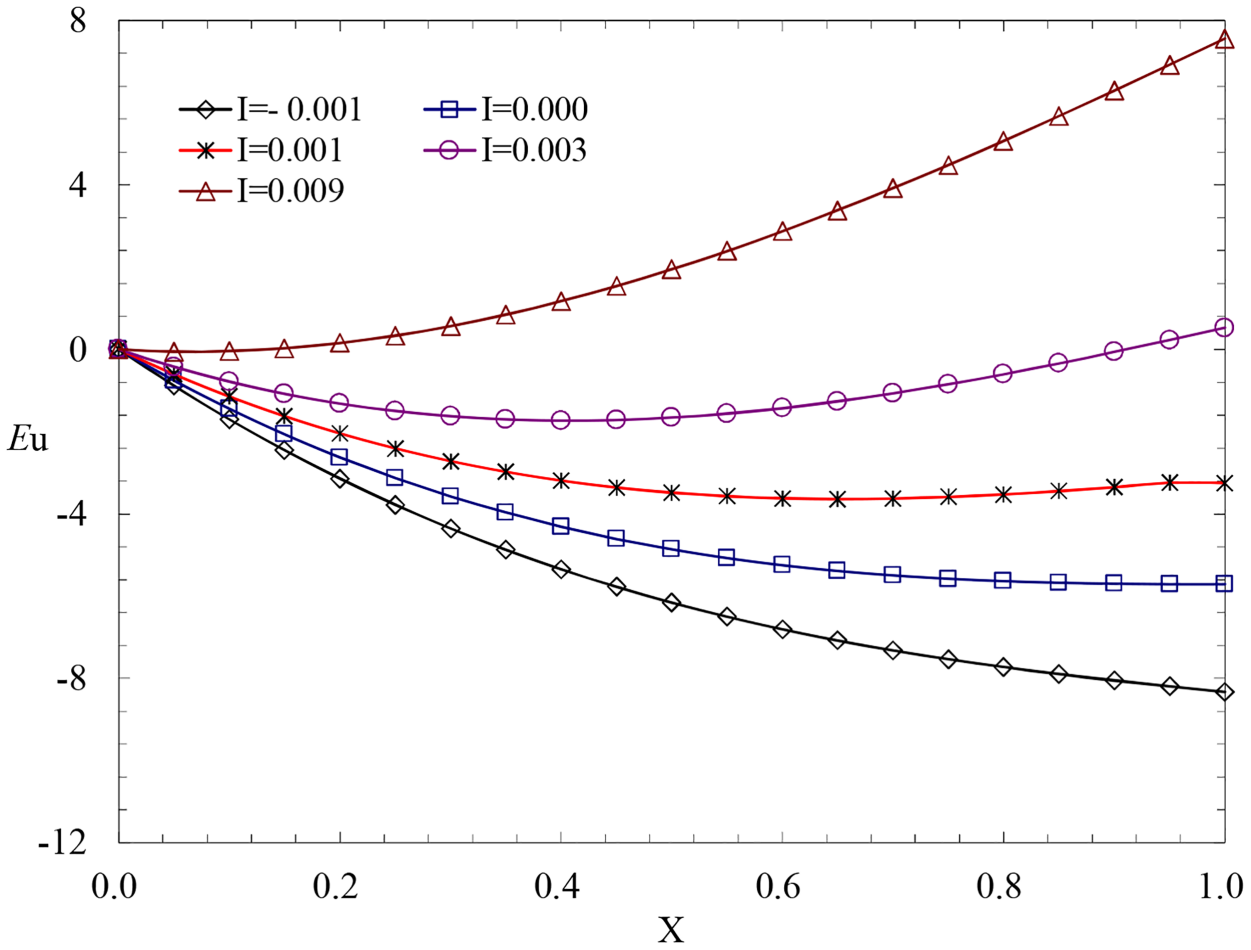
Effects of the slope on the pressure profiles.

In the analytical solution of the variable mass momentum equation, the gravity item reflects the influence of the slope *I* on the value of *Eu*. For the condition in which the diameter of the pipe *D* = 0.04775m, the length of the pipe *L* = 50m, the interval of the holes *S* = 0.15m, the diameter of the holes *d* = 0.0012m, and the water head at the entrance *H* = 0.5m, the curve of *I*~*Eu* has been calculated and is presented in Fig 4. In Fig 4, the length-diameter *E* = 1047 and the range of the flow parameter*R*e_0_lies in the range of 20538–28049. According to the calculated results, in this condition, the influence of the friction item is larger than that of the momentum item because of the minor velocity. Thus, the perforated pipe flow is friction-controlled flow in different slopes, and the pressure distribution is controlled by gravity and friction.

According to Fig 4, if*I*≤0, the hydrodynamic pressure decreases along the flow because of the double effect of gravity and friction and reaches a maximum at the entrance and a minimum at the closed end. If *I*>0, the gravity item is positive, which is opposite to the friction. If *I* = 0.003, the force of gravity is counteracted by the friction, and in this condition, the hydrodynamic pressure tends to be uniform along the flow. With an increase in slope *I*, the force of gravity exceeds the friction, the sign of *Eu* changes from negative to positive; thus, the maximum of the hydrodynamic pressure occurs at the closed end.

### Influence of length-diameter on the pressure distribution

**Fig 5:**
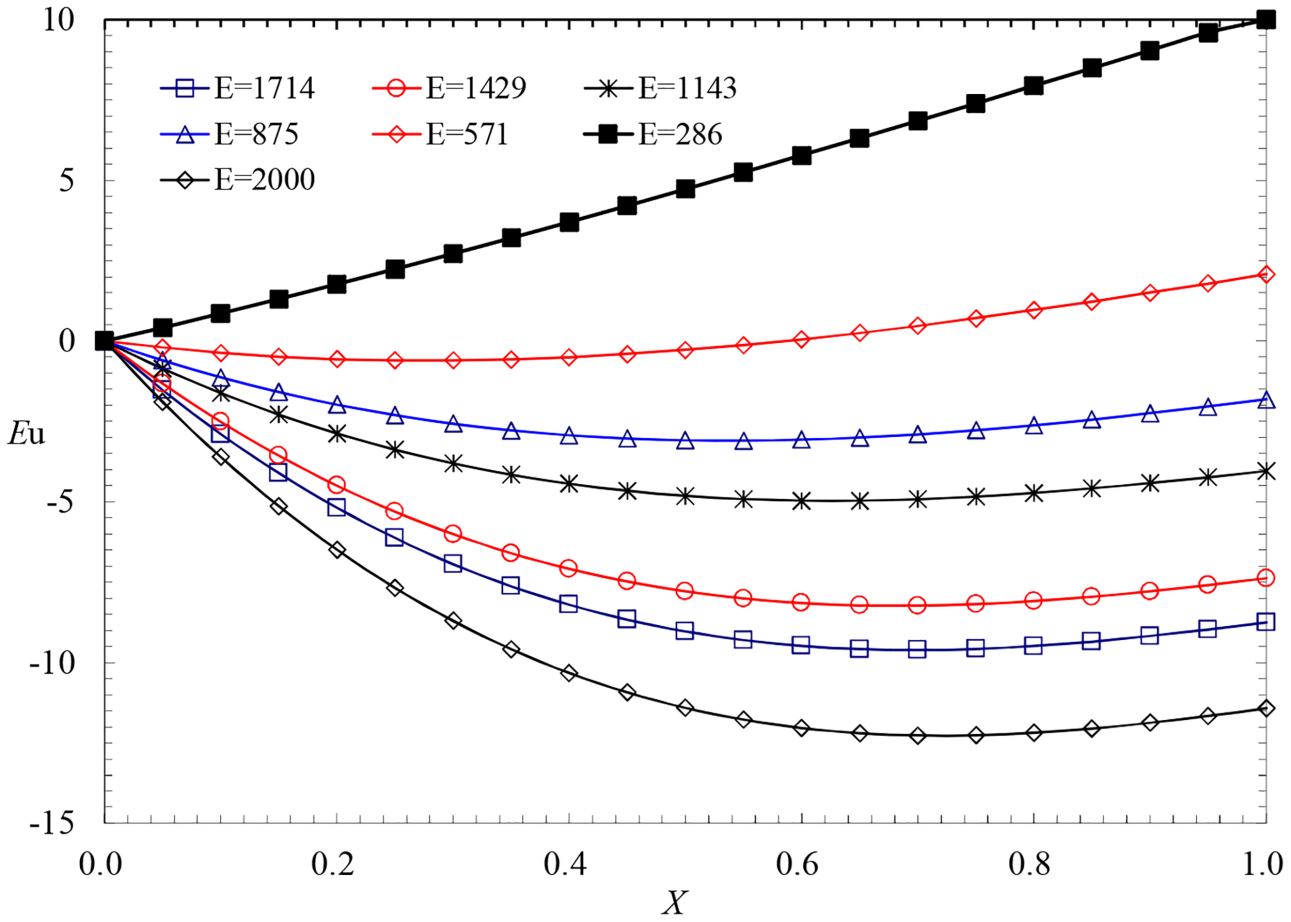
Effects of the ratio of length to diameter on the pressure profiles.

If the diameter of the pipe *D* = 0.035m, the slope *I* = 0.001, the interval of the holes *S* = 0.3m, the diameter of the holes *d* = 0.0012m, and the water head at the entrance *H* = 0.5m, the calculated results of Eq (16) with different values of *E* are shown in Fig 5.

From Fig 5, we observe that if *E*≥571, the pressure distribution curve has a downward concave shape with the minimum value at the point *X*. Before this point, the force of the friction item for *Eu* is greater than the gravity and momentum items; thus, both *Eu* and the hydrodynamic pressure decrease along the flow. At the point, the influences of gravity, momentum and friction are balanced, and the hydrodynamic pressure reaches a minimum. After the point, the force of the friction item for *Eu* is less than that of the gravity and momentum items; thus, *Eu* increases along the flow, and the hydrodynamic pressure increases along the flow. If *E* = 700, the influences are balanced. In this condition, the hydrodynamic pressure is uniform along the flow. If *E* = 286,*Eu* increases linearly along the flow, which indicates that in this condition, the forces of momentum and friction are balanced and the pressure distribution of the porous pipe increases linearly with the gravity item. When *E* decreases, the influence of momentum is greater than the friction, and the pressure distribution curve has an upward concave shape. In this condition, the porous pipe flow is momentum-controlled flow, which always appears in the chemical field.

### Comparison of measured and predicted results

#### Comparison of measured and predicted results under different slopes

**Fig 6:**
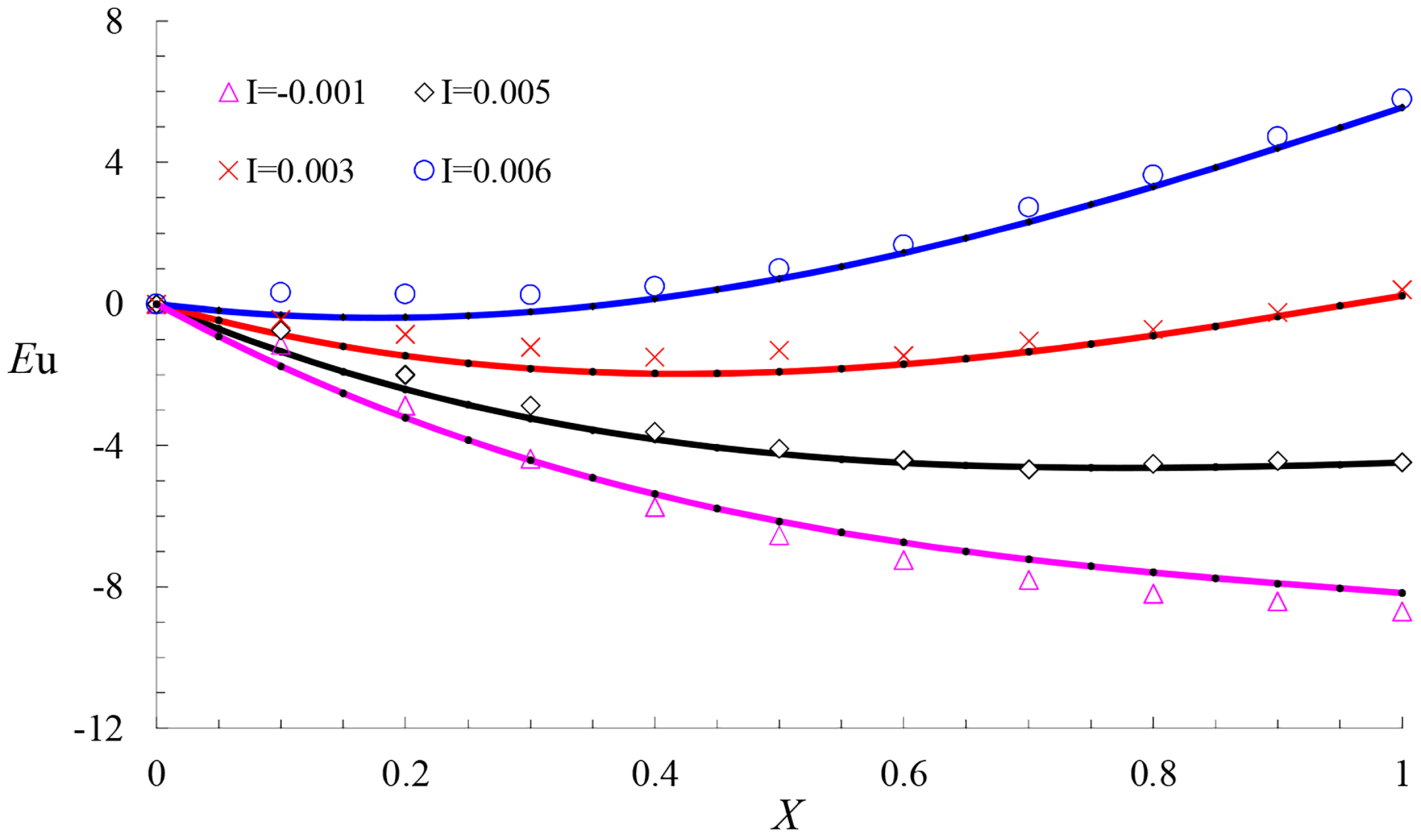
The comparison between the calculated results and experimental values.

If diameter of the pipe *D* = 0.04775m, the length of the pipe *L* = 50m (*E* = 1047), the interval of the holes *S* = 0.15m, the diameter of the holes *D* = 0.0012m and the water head at the entrance *H* = 0.5m, the exponent *z* in different conditions of the slope can first be calculated according to the Eq (6), and the variation of *Eu* can be calculated according to Eq (16). Finally, the comparison between the predicted and experimental results is given in Fig 6. Considering the minor velocity (the velocity at the entrance*V*_0_≤0.6m/s) and the limited experimental condition, the minor head difference between the start and the entrance (which causes a larger deviation with the maximum relative error is 74.27%), and the analytical results from the calculation and the experimental results are identical with the minimum relative error is 0.02%, which indicates that the porous distribution of the power rate assumption is reasonable.

### Comparison of measured and predicted results under different length-diameter ratios

**Fig 7:**
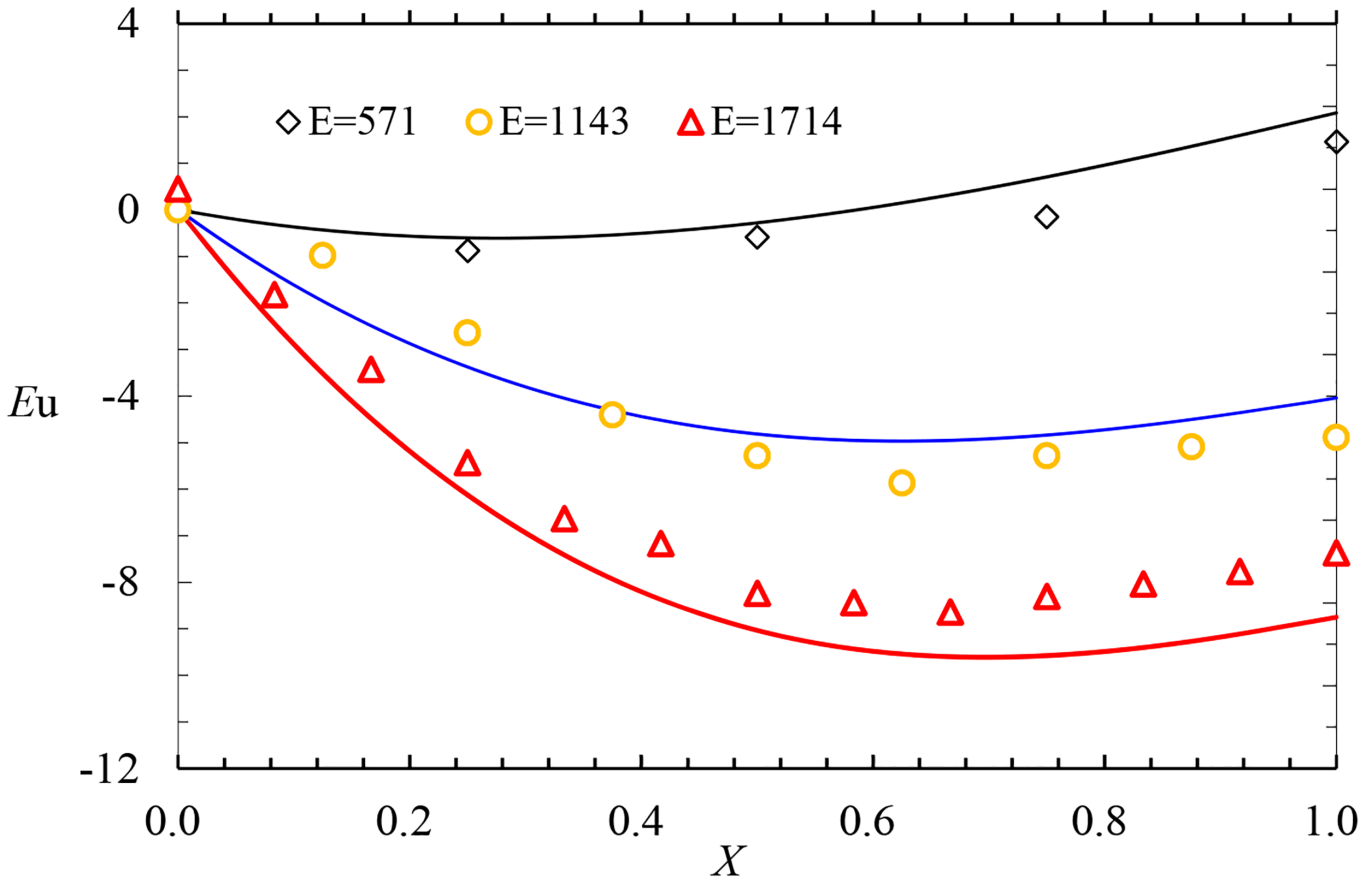
Comparison between the predicted results and experimental values.

Under the experimental condition in which the diameter of the pipe *D* = 0.035m, the length of the pipe *I* = 0.001, the interval of the holes *S* = 0.3m, the diameter of the holes *d* = 0.0012m and *H* = 0.5m, the comparisons between the predicted and experimental results in three sets of conditions of *E* are shown in Fig 7. The experimental results indicate that the results obtained for the calculation and experiments are identical.

**Fig 8:**
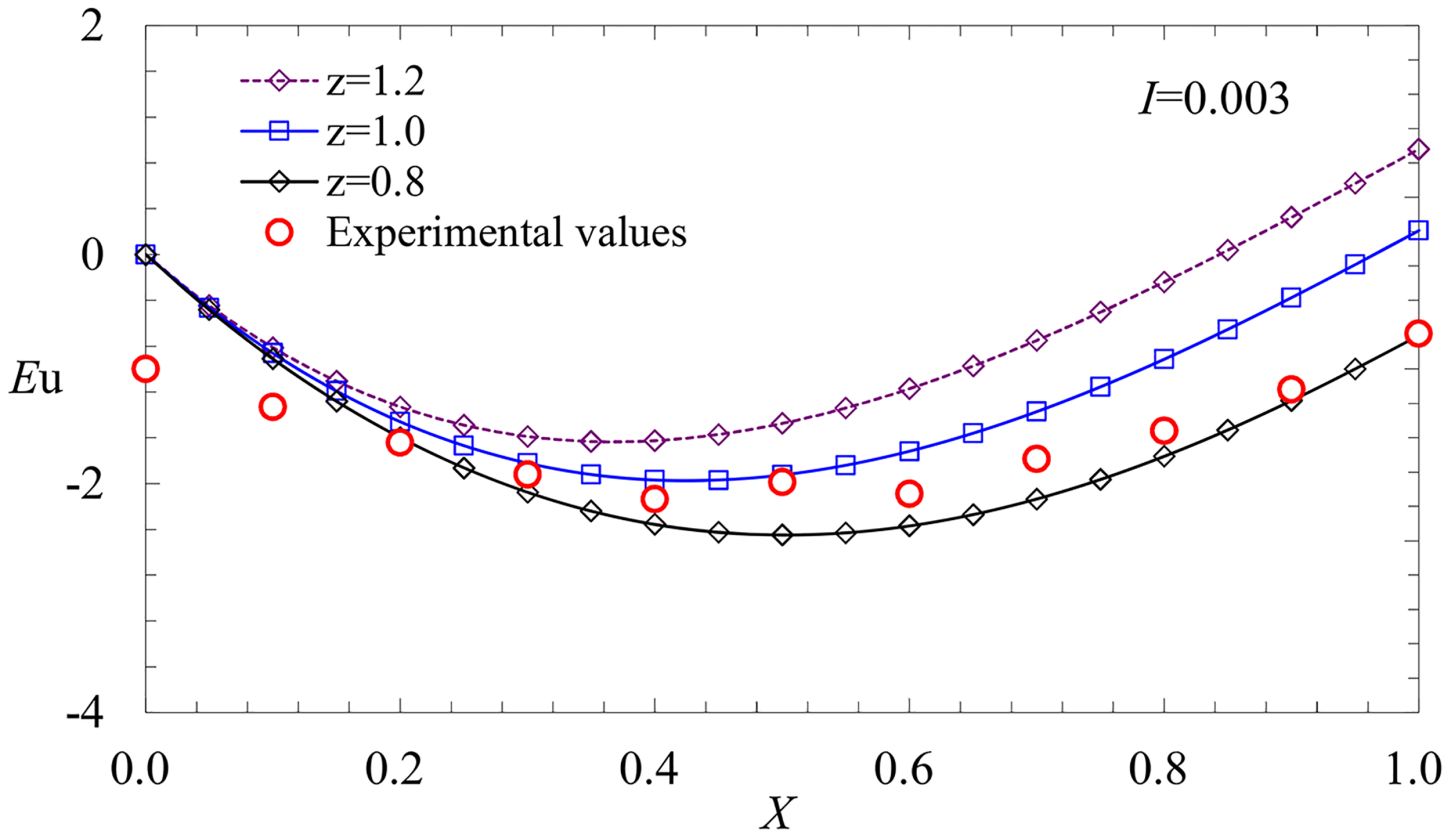
Effects of *z* on the pressure profiles.

The analytical solution contains two parameters that can affect the results of the pressure distribution. One parameter is the exponent *z* of the velocity distribution, and the other is the modified coefficient *χ* of the drag coefficient. For *z* = 0.8,1,1.2, a comparison between the predicted and experimental results is shown in Fig 8.

From Fig 8, we observe that the variation of the exponent *z* has an effect on the pressure distribution, especially at the closed end. Generally speaking, the deviation of the exponent *z* from 1.0 is larger, and the influence of *z* is larger, which is consistent with the experimental results. According to Eq (16), the modified coefficient *χ* also has a greater effect on the closed end. The selection of the two parameters according to experimental system is a critical problem that should receive additional attention.

#### Comparison with other experimental results

The perforated flow distributors in the chemical field and the perforated pipes in the water supply and drainage field are characterized by the slope *I* = 0, the minor length-diameter ratio *E* and the opening ratio *η* = *Nd*^2^/(4*DL*)(*η*>>*η*_0_). In this condition, *z*>0 can be derived according to Eq (6), which is obviously unreasonable. For these types of pipes, the variation law of the exponent *z* with the opening ratio *η* should be cautiously investigated. To simplify the calculation, *α* = 0.65,*β* = 0.15, *z* = 1 and *χ* = 1.3 are selected.

**Fig 9:**
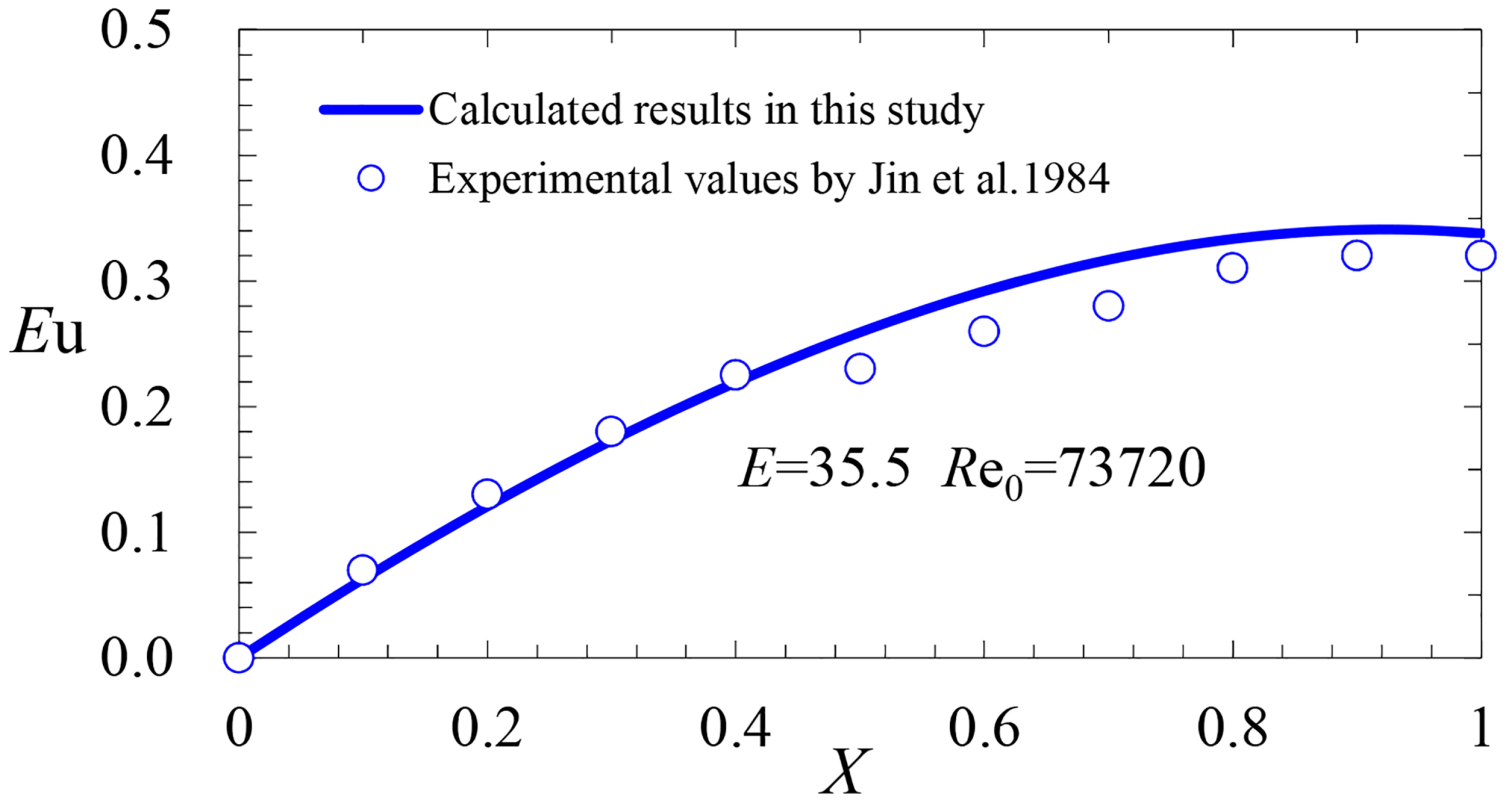
Comparison between the calculated results and experimental values by Jin et al. 1984.

A comparison between the predicted results calculated by Eq (16) and the experimental values measured by Jin et al [4] is shown in Fig 9 (*D* = 25.6mm, *L* = 910mm, *d* = 6mm, *S* = 91mm, *N* = 10, *E* = 35.5 and *R*e_0_ = 73720). The mean error between the calculated results and the experimental values is 8.2%.

**Fig 10:**
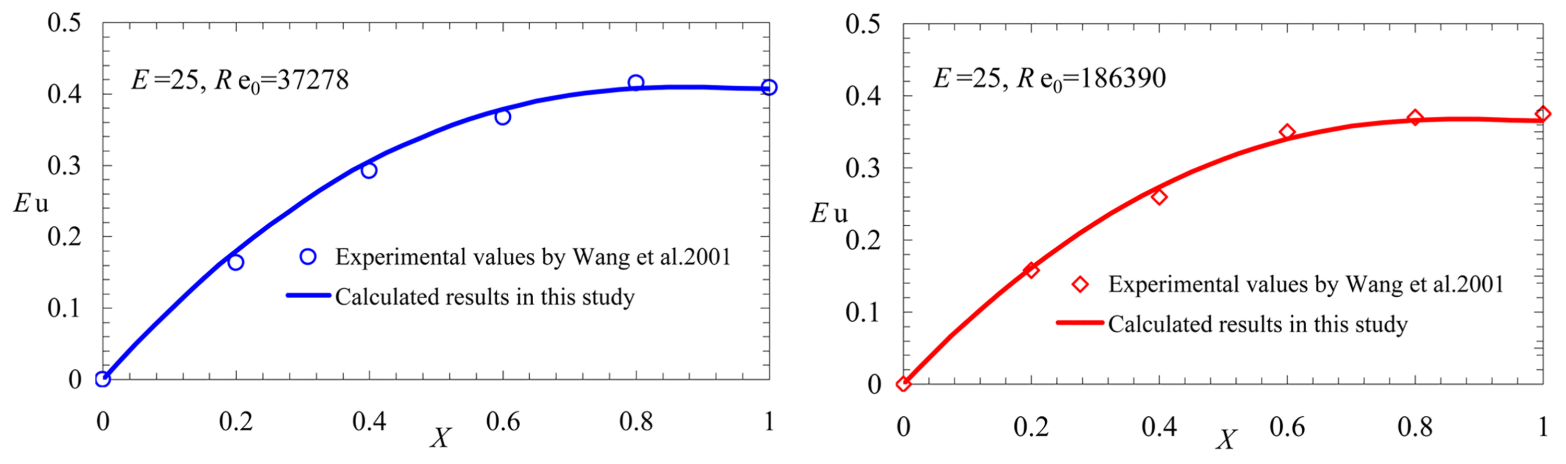
Comparison between the calculated results and experimental values by Wang et al. 2001.

Comparisons between the predicted results calculated according to Eqs (16) and (18) and the experimental values from Wang et al [8] are separately illustrated in Fig 10 (*D* = 21mm,*L* = 525mm, *d* = 3mm, *N* = 21 and *E* = 25). The mean errors between the calculated results and the experimental values are 3.8% and 2.7%, respectively, which showed that the analytical solution of the variable mass momentum equation was qualitatively and quantitatively consistent with the experimental results.

The comparison shows that the results from the calculation and the experiments are identical, which indicates that the analytical solution of the variable mass momentum equation can be applied widely and is suitable for both friction- and momentum-controlled flow, which also appears in the chemical field.

For momentum-controlled flow, the friction item is less than the momentum item, the force of the momentum is greater than that of the friction, and the pressure of the porous pipe thus increases along the flow. According to *I* = 0 and Eq (16b), we obtain

$${Eu|}_{X=1}=\;\alpha \;-\;\beta -\;\frac{0.1582\chi E}{(1.75z+\;1)\;{\text{Re}}_{0}{}^{0.25}}=0.5-\frac{0.1582\chi E}{(1.75z+\;1)\;{\text{Re}}_{0}{}^{0.25}}$$ (19)

From Eq (19), we note that *E*_*u*_|_*X* = 1_<0.5 for the momentum-controlled flow, and the analytical results of Figs 9 and 10 are consistent with this view.

## Conclusions

(i) According to the momentum law, the momentum equation of the variable mass, which depends on the momentum exchange coefficient *k* and the drag coefficient *λ*, is established in the condition of uniform slope.

(ii) The velocity distribution is analyzed according to the perforated distribution of the power rate assumption, and deduction of the empirical formula for the exponent *z* is based on the experimental data. In the process of engineering design, we can adjust the slope, the length-diameter ratio, and the opening ratio of the porous pipe to make the exponent *z* tend toward 1 and reduce the relative deviation of the discharge. Thus, we can ensure the uniformity of different holes.

(iii) We analyze the variation laws of the drag coefficient *λ* and the momentum exchange coefficient *k* and deduce the function relationship of *k*. The function relationship contains two constant parameters. One parameter is *α*, which denotes the momentum principle of the first hole, and*α*≌0.65. The other parameter is (*α*−*β*), which indicates the momentum principle of the last hole and (*α*−*β*) = 0.5 and *β*≌0.15 at the closed end.

(iv) Analysis of the momentum equation indicates that the major forces for the porous pipe are hydrodynamic pressure, gravity and friction. These three forces are interrelated and impact the perforated flow. The analytical solution is composed of the components of pressure, the gravity, friction and momentum, which reflect the comprehensive influence of the factors. In the process of engineering design, if the slope given, we can adjust the length-diameter and Reynolds numbers to balance the influences of gravity, momentum and friction. In this condition, the hydrodynamic pressure distribution tends toward a uniform distribution, and the discharge from the different holes is the same.

(v) Verifications from previous studies and the experimental data indicate that the analytical results from the calculation and the results from the experiments are identical which the mean errors are 8.2%, 3.8% and 2.7%. This outcome shows that the analytical solution of the variable mass momentum equation can be applied widely and is suitable for both friction-controlled flow, which is influenced by gravity and friction, and momentum-controlled flow, which is influenced by momentum and friction.
